# Correction: Electrochemical investigation and amperometry determination iodate based on ionic liquid/polyoxotungstate/P-doped electrochemically reduced graphene oxide multi-component nanocomposite modified glassy carbon electrode

**DOI:** 10.1039/d1ra90108g

**Published:** 2021-05-11

**Authors:** Minoo Sharifi, Somayeh Dianat, Amin Hosseinian

**Affiliations:** Department of Chemistry, Faculty of Sciences, University of Hormozgan Bandar Abbas 79161-93145 Iran s.dianat@hormozgan.ac.ir +98 76 33670121

## Abstract

Correction for ‘Electrochemical investigation and amperometry determination iodate based on ionic liquid/polyoxotungstate/P-doped electrochemically reduced graphene oxide multi-component nanocomposite modified glassy carbon electrode’ by Minoo Sharifi *et al.*, *RSC Adv.*, 2021, **11**, 8993–9007, DOI: 10.1039/D1RA00845E.

The authors regret that eqn (3) and (4) were shown incorrectly in the original article.

The corrected equations are shown below.

3H_4_SiW_4_^V^W_7_^VI^Ni(H_2_O)O_39_^6−^ + IO_3_^−^ → 3H_2_SiW_2_^V^W_9_^VI^Ni(H_2_O)O_39_^6−^ + I^−^ + 3H_2_O (3)

3H_6_SiW_6_^V^W_5_^VI^Ni(H_2_O)O_39_^6−^ + IO_3_^−^ → 3H_4_SiW_4_^V^W_7_^VI^Ni(H_2_O)O_39_^6−^ + I^−^ + 3H_2_O (4)

The Royal Society of Chemistry apologises for these errors and any consequent inconvenience to authors and readers.

## Supplementary Material

